# Stable long-term outcomes after cochlear implantation in subjects with *TMPRSS3* associated hearing loss: a retrospective multicentre study

**DOI:** 10.1186/s40463-023-00680-3

**Published:** 2023-12-15

**Authors:** M. L. A. Fehrmann, W. J. Huinck, M. E. G. Thijssen, L. Haer-Wigman, H. G. Yntema, L. J. C. Rotteveel, J. C. C. Widdershoven, T. Goderie, M. F. van Dooren, E. H. Hoefsloot, M. P. van der Schroeff, E. A. M. Mylanus, DOOFNL consortium, M. F. van Dooren, S. G. Kant, H. H. W. de Gier, E. H. Hoefsloot, M. P. van der Schroeff, L. J. C. Rotteveel, F. G. Ropers, M. Kriek, E. Aten, J. C. C. Widdershoven, J. R. Hof, K. Hellingman, V. Vernimmen, H. Kremer, R. J. E. Pennings, I. Feenstra, C. P. Lanting, H. G. Yntema, F. L. J. Cals, L. Haer-Wigman, R. H. Free, J. S. Klein Wassink-Ruiter, A. L. Smit, M. J. van den Boogaard, A. M. A. Lachmeier, J. J. Smits, F. A. Ebbens, S. M. Maas, A. Plomp, T. P. M. Goderie, P. Merkus, J. van de Kamp, C. P. Lanting, R. J. E. Pennings

**Affiliations:** 1 Department of Otorhinolaryngology, Radboudumc, Nijmegen, The Netherlands; 2 Department of Clinical Genetics, Radboudumc, Nijmegen, The Netherlands; 3 Department of Otorhinolaryngology, Leiden UMC, Leiden, The Netherlands; 4 Department of Otorhinolaryngology, Maastricht UMC, Maastricht, The Netherlands; 5 Department of Otorhinolaryngology-Head and Neck Surgery, Ear and Hearing, Amsterdam UMC, Amsterdam, The Netherlands; 6 Department of Clinical Genetics, Erasmus MC, Rotterdam, The Netherlands; 7 Department of Otorhinolaryngology, Erasmus MC, Rotterdam, The Netherlands

**Keywords:** Cochlear implantation, Hereditary hearing loss, Sensorineural hearing loss, *TMPRSS3*, Cochlear implant outcomes, Clinical decision-making, Disease management

## Abstract

**Background:**

The spiral ganglion hypothesis suggests that pathogenic variants in genes preferentially expressed in the spiral ganglion nerves (SGN), may lead to poor cochlear implant (CI) performance. It was long thought that TMPRSS3 was particularly expressed in the SGNs. However, this is not in line with recent reviews evaluating CI performance in subjects with *TMPRSS3-*associated sensorineural hearing loss (SNHL) reporting overall beneficial outcomes. These outcomes are, however, based on variable follow-up times of, in general, 1 year or less. Therefore, we aimed to 1. evaluate long-term outcomes after CI implantation of speech recognition in quiet in subjects with *TMPRSS3-*associated SNHL, and 2. test the spiral ganglion hypothesis using the *TMPRSS3-*group.

**Methods:**

This retrospective, multicentre study evaluated long-term CI performance in a Dutch population with *TMPRSS3*-associated SNHL. The phoneme scores at 70 dB with CI in the *TMPRSS3*-group were compared to a control group of fully genotyped cochlear implant users with post-lingual SNHL without genes affecting the SGN, or severe anatomical inner ear malformations. CI-recipients with a phoneme score ≤ 70% at least 1-year post-implantation were considered poor performers and were evaluated in more detail.

**Results:**

The *TMPRSS3 group* consisted of 29 subjects (N = 33 ears), and the control group of 62 subjects (N = 67 ears). For the *TMPRSS3*-group, we found an average phoneme score of 89% after 5 years, which remained stable up to 10 years post-implantation. At both 5 and 10-year follow-up, no difference was found in speech recognition in quiet between both groups (*p* = 0.830 and *p* = 0.987, respectively). Despite these overall adequate CI outcomes, six CI recipients had a phoneme score of ≤ 70% and were considered poor performers. The latter was observed in subjects with residual hearing post-implantation or older age at implantation.

**Conclusion:**

Subjects with *TMPRSS3*-associated SNHL have adequate and stable long-term outcomes after cochlear implantation, equal to the performance of genotyped patient with affected genes not expressed in the SGN. These findings are not in line with the spiral ganglion hypothesis. However, more recent studies showed that TMPRSS3 is mainly expressed in the hair cells with only limited SGN expression. Therefore, we cannot confirm nor refute the spiral ganglion hypothesis.

**Supplementary Information:**

The online version contains supplementary material available at 10.1186/s40463-023-00680-3.

## Background

Hearing loss is one of the most common and frequently diagnosed sensory disorders, with 50–70% of cases attributable to genetic causes [1]. Currently, more than 120 genes have been identified to be associated with non-syndromic hearing loss [2]. *TMPRSS3* is one of these genes and encodes for a type II transmembrane serine protease. Pathogenic variants in *TMPRSS3* cause autosomal recessively inherited sensorineural hearing loss (SNHL) that accounts for 0.7% up to 11% of cases with autosomal recessive NSHL, depending on the geographic origin [3]. *TMPRSS3-*associated SNHL may present with congenital severe-to-profound SNHL or post-lingual onset high-frequency (sloping) SNHL with relatively unaffected hearing at the lower frequencies [4]. Rehabilitation depends on the type and severity of SNHL.

Cochlear implantation (CI) outcomes in subjects with pathogenic variants in *TMPRSS3* have been reported in multiple studies, showing inconsistent outcomes [5–12]. Eppsteiner et al. reported on two poor CI performers with *TMPRSS3*-associated hearing loss and concluded that pathogenic variants in genes expressed in the spiral ganglion neurons (SGN) or in the auditory nerve, negatively affect CI outcomes. According to the spiral ganglion hypothesis, poor CI performance is expected when the SGNs and/or auditory nerves degenerate over time, while good CI performance is anticipated when only the hair cells (HCs) are affected [8]. Three recent studies reviewed the literature on CI performance in *TMPRSS3*-associated SNHL based on an almost identical set of publications [3, 13, 14]. These studies all concluded that cochlear implantation is a beneficial intervention. However, heterogeneous outcome measures made comparisons difficult, and conclusions were based on varying follow-up times of, in general, 1 year or less. The latter still does not rule out long-term deterioration of function.

Although previous studies reported Tmprss3 expression in SGNs in mice [15, 16], Chen et al. demonstrated that Tmprss3 is highly expressed in HCs with only limited SGN expression in mice [14]. A highly specific expression of TMPRSS3 in HCs was also observed in human inner ear organoids [13]. These findings suggest that *TMPRSS3*-associated SNHL might be the consequence of dysfunctional HCs and not due to dysfunctional SGNs. Chen et al. further showed that pathogenic variants in *Tmprss3* result in rapid HC degeneration, causing delayed-onset progressive SGN degeneration [14]. This makes it especially interesting to evaluate long-term CI outcomes in subjects with *TMPRSS3-*associated SNHL since these findings may indicate that CI performance will deteriorate over time. The aims of this study were to 1. present the results of long-term CI performance in a large Dutch population of subjects with *TMPRSS3-*associated SNHL, and 2. to evaluate the spiral ganglion hypothesis using the outcomes of these subjects.

## Methods

### Study design and population

This retrospective, observational, multicentre cohort study evaluated CI performance in CI recipients with *TMPRSS3*-associated SNHL. The Radboud University Medical Centre assembled a study cohort with genotyped CI recipients. Subjects were included in this cohort when they 1. had a confirmed genetic diagnosis based on monoallelic or biallelic (likely) pathogenic variants in respectively dominant or recessive inherited genes associated with SNHL; 2. received a cochlear implant between 1996 and 2021; 3. had at least 1-year of follow-up measurements of the speech recognition. Subjects were excluded from this study when aged ≥ 70 years at implantation, or when they had SNHL related to other causes, i.e., prenatal TORCH (toxoplasmosis, rubella, CMV, HSV) infections, aminoglycoside exposure, otoacoustic trauma, meningitis, or hyperbilirubinemia. The *TMPRSS3*-subjects were selected from this study cohort, and additional subjects were recruited from the other academic centres in the Netherlands that are part of the DOOFNL consortium. A *TMPRSS3*-group was created and included subjects with a confirmed genetic diagnosis based on biallelic (likely) pathogenic variants in *TMPRSS3* with at least 1 year of follow-up measurements of speech recognition scores. Subjects with at least 5 years of follow-up were separately evaluated to objectify long-term CI performance and were compared to the long-term outcomes of a control group. This control group was created from the same study cohort of genotyped CI recipients from the Radboud University Medical Centre by enrolling subjects with a confirmed genetic diagnosis of postlingual SNHL. Subjects with pathogenic variants in genes known to affect the spiral ganglion neurons or auditory nerve (e.g., *OPA1* and *OTOF*) were excluded from the control group, as were subjects with severe cochleovestibular abnormalities on imaging. Subjects with an enlarged vestibular aqueduct (EVA) were not excluded from the control group because these subjects have progression of SNHL in the same age segment as the *TMPRSS3-*group. Additionally, previous studies categorized EVA as the most subtle detectable inner ear malformation [17, 18]. Moreover, CI outcomes and surgery-related complications are comparable in recipients with an EVA and without inner ear malformations [19–21].

### Data collection

Demographic factors were collected by chart review and included gender, age of onset of SNHL, use of hearing aids, learning difficulties, and age at time of implantation. All pre- and postoperative audiovestibular examinations were evaluated. Vestibular testing was performed by calorisation, and rotatory chair, using electronystagmography (ENG). Furthermore, the video head impulse test (vHIT) was used to assess bilateral semicircular canal function. Results of imaging were included to assess cochleovestibular abnormalities. The surgical approach and side of implantation were collected to evaluate surgical factors. The type of implant and electrode (Lateral wall- or peri-modiolar electrode) were also recorded. The genetic diagnosis was gathered by scoring the variant(s) with the associated protein change(s), affected domain(s), type of variant (truncating or missense), and classification (according to the AMG association guidelines [22]). No additional genetic analyses or audiological tests were performed.

Hearing was evaluated by standard pure tone and speech audiometry according to current standards. Phoneme scores were presented at 70 dB HL in quiet and were assessed both aided and unaided. The pure tone average (PTA) was calculated using thresholds at 500, 1000, 2000, and 4000 Hz (PTA_0.5–4kHz_). In the *TMPRSS3* group, not all subjects used hearing aids prior to implantation because of significant residual hearing at the lower frequencies. We assessed the best-aided/unaided-PTA and -phoneme scores to compare the pre-implantation hearing performance with the performance post-implantation. These best-aided/unaided scores were calculated from aided scores from subjects using hearing aids prior to implantation and combined with the unaided scores from subjects not using hearing aids prior to implantation. Where aided scores from subjects using hearing aids were not available, unaided scores were used. Residual hearing preservation (HP) post-implantation was defined by the Hearing Preservation Classification System as reported by Skarzynski et al. [23]. To calculate the percentage of residual HP (HP%), the following formula was used:

$$HP \left(\%\right)=100 \times \left(1-\left(\frac{{PTA}_{post}-{PTA}_{pre}}{120-{PTA}_{pre}}\right)\right)$$

An HP% > 75% was classified as complete HP, HP% > 25–75% as partial HP, and HP% 0–25% as minimal HP. CI-recipients with a phoneme score ≤ 70% at least 1-year post-implantation were considered poor performers and were evaluated in more detail.

### Data analysis

Statistical analyses were performed with IBM Statistical Package for the Social Science Statistics (SPSS).

A Chi-squared test was used to compare categorical data (side implanted ear, hearing aid prior to implantation, surgical approach, and affected genes) between the *TMPRSS3* group and the control group, while the mean age at implantation, self-reported duration of hearing loss, PTA, and phoneme scores between these groups were compared using the Mann–Whitney U test. This test was also used to compare phoneme scores and HP% between different types of electrodes. The mean PTA and phoneme scores at other follow-up moments within the *TMPRSS3* group were compared using the Wilcoxon signed-rank test. The Kruskal–Wallis test was used to compare the mean PTA, phoneme scores, and HP% between the different surgical approaches. Univariate regression analysis was performed to study the correlation between residual hearing post-implantation and non-/limited CI use. The same analysis was performed to test whether the age of implantation correlated with the postimplantation phoneme scores. A multiple regression analysis was used to further assess this correlation while correcting for confounders. The Pearson correlation coefficient was used for multicollinearity testing. A *p* value < 0.05 was considered statistically significant.

## Results

### Subjects and surgical procedure

After evaluation of in- and exclusion criteria, 27 subjects with bi-allelic pathogenic *TMPRSS3* variants were included in the *TMPRSS3* group. In 33 ears, cochlear implantation was performed (Tables 1, 2). A considerable variation in the self-reported age of onset was found. All subjects reported progressive bilateral SNHL. Twelve ears were not rehabilitated with hearing aids prior to cochlear implantation (36.4%). These twelve subjects tried hearing aids but reported little to no benefit. Furthermore, the mean preoperative unaided PTA_0.5-4kHz_ was significantly lower in these twelve subjects (*P* = 0.024), see Table 3. Imbalance was reported by only one subject (B1). The surgical approach was split almost evenly between a cochleostomy (46%) and a round window insertion (49%). The implanted devices and electrode arrays are shown in Table 2. The control group consisted of 62 subjects, in which a total of 67 ears were implanted (Table 1). The choice of surgical technique significantly differed between the *TMPRSS3* group and the control group (*p* = 0.002) as in the first group, we aimed to preserve residual low-frequency hearing. Further, the number of EVAs was significantly higher in the control group (*p* = 0.045).

**Table 1:** Patient characteristics

Characteristic	TMPRSS3-Group, N = 33 ears (100%)	Control-group, N = 67 ears (100%)	*P* value
Gender, % female	15 (45.5)	43 (64.2)	0.074
Age at implantation (mean ± SD)	24 ± 19	27 ± 26	0.584
Duration of hearing loss prior to implantation (mean ± SD)	16 ± 14	17 ± 18	0.908
Learning difficulties	1 (3.0)		0.165
EVA on CT or MRI	0 (0.0)	15 (22.4)	0.045
Affected gene			
ACTB	0 (0.0) (0 (0.0).0 (0.0))	1 (1.5)	
ACTG1	0 (0.0) (0 (0.0).0 (0.0))	1 (1.5)	
ADGRV1	0 (0.0)	1 (1.5)	
CEP95	0 (0.0)	1 (1.5)	
CLRN1	0 (0.0)	3 (4.5)	
COCH	0 (0.0)	10 (14.9)	
GJB2	0 (0.0)	8 (11.9)	
GJB6	0 (0.0)	1 (1.5)	
LARS2	0 (0.0)	1 (1.5)	
MITF	0 (0.0)	2 (3.0)	
MITO	0 (0.0)	1 (1.5)	
MYO15A	0 (0.0)	5(7.5)	
MYO7A	0 (0.0)	4 (6.0)	
POU4F3	0 (0.0)	1 (1.5)	
PRPS1	0 (0.0)	1 (1.5)	
PTPN11	0 (0.0)	1 (1.5)	
SLC26A4	0 (0.0)	15 (22.4)	
SOX10	0 (0.0)	1 (1.5)	
TMPRSS3	33 (100)	0(0.0)	
TPRN	0 (0.0)	2 (3.0)	
TUBB4B	0 (0.0)	2 (2.5)	
USH2A	0 (0.0)	3 (4.5)	
WFS1	0 (0.0)	2 (3.0)	
CI side			
Left	15 (45.5)	30 (44.8)	0.173
Right	14 (42.4)	35(52.2)	
Bilateral (simultaneously)	4 (21.1)	2 (3.0)	
Hearing aid in ear to be implanted	21 (63.6)	46 (68.7)	0.616
Surgical technique			
Cochleostomy	15 (45.5)	54 (80.6)	0.002
Round window	16 (48.5)	13 (19.4)	
Extended round window	1 (3.0)	0 (0.0)	
Not reported	1 (3.0)	0 (0.0)	

*SD* standard deviation, *EVA* enlarged vestibular aqueduct, CT computer tomography, *MRI* magnetic resonance imaging, *CI* cochlear implant

**Table 2:** Patient characteristics TMPRSS3-Group

^Patient*^	^Gender^	^Age at implantation^	^cDNA variant 1**^	^Protein variant 1^	^cDNA variant 2**^	^Protein variant 2^	^Self-reported duration of HL prior to implantation^	^Self-reported age of onset HL^	^Degree HL at time of implantation***^	^Vestibular function in ear to be implanted****^	^Reported balance problems prior to implantation^	^Hearing aid in ear to be implanted^	^Implanted ear^	^Implanted device^
^A1^	^F^	^10^	^c.413C>A^	^p.(Ala138Glu)^	^c.916G>A^	^p.(Ala306Thr)^	^7.5^	^2.5^	^Severe^	^Normal^	^−^	^+^	^Right^	^CI24RE (ST)^
^B1^	^F^	^25^	^c.208del^	^p.(his70fs)^	^c.1276G>A^	^p.(Ala426Thr)^	^12^	^13^	^Profound^	^Hyporeflexia^	^+^	^+^	^Right^	^CI24RE (CA)^
^C1−1^	^F^	^4^	^c.208del^	^p.(his70fs)^	^c..916G>A^	^p.(Ala306Thr)^	^1^	^3^	^Severe^	^Normal^	^−^	^+^	^Left^	^CI24RE (CA)^
^C1−2^		^13^					^10^		^Profound^	^Normal^		^+^	^Right^	^CI512^
^D1−1^	^F^	^5^	^c.595G>A^	^p.(Val199Met)^	^c.936del^	^p.(Pro313fs)^	^2.5^	^2.5^	^Profound^	^Normal^	^−^	^+^	^Links^	^CI24RE (ST)^
^D1−2^									^Profound^	^Normal^		^+^	^Right^	^CI24RE (ST)^
^E1^	^M^	^17^	^c.413C>A^	^p.(Ala138Glu)^	^c.595G>A^	^p.(Val199Met)^	^13^	^4^	^Moderate^	^Normal^	^−^	^+^	^Right^	^CI422^
^F1−1^	^M^	^6^	^c.413C>A^	^p.(Ala138Glu)^	^c.595G>A^	^p.(Val199Met)^	^3.5^	^2.5^	^Profound^	^Normal^	^−^	^+^	^Right^	^CI522^
^F1−2^		^9^					^6.5^		^Profound^	^Normal^		^+^	^Left^	^CI24RE (ST)^
^G1^	^F^	^52^	^c.413C>A^	^p.(Ala138Glu)^	^c.916G>A^	^p.(Ala306Thr)^	^44^	^8^	^Profound^	^Normal^	^−^	^+^	^Left^	^CI24RE (CA)^
^H1^	^M^	^29^	^c.413C>A^	^p.(Ala138Glu)^	^c.916G>A^	^p.(Ala306Thr)^	^17^	^12^	^Profound^	^Hyporeflexia^	^−^	^+^	^Right^	^CI522^
^I1^	^M^	^16^	^c.413C>A^	^p.(Ala138Glu)^	^c.595G>A^	^p.(Val199Met)^	^<12^	^<4^	^Moderate^	^Normal^	^−^	^−^	^Right^	^CI632^
^J1−1^	^M^	^4^	^c.916G>A^	^p.(Ala306Thr)^	^c.280G>T^	^p.(Gly94*)^	^NA^	^<4^	^Profound^	^NA^	^−^	^−^	^Left^	^CI632^
^J1−2^									^Profound^				^Right^	^CI632^
^K1^	^F^	^46^	^c.325C>T^	^Arg109Trp^	^c.1276G>A^	^p.(Ala426Thr)^	^20^	^26^	^Profound^	^Normal^	^−^	^+^	^Left^	^CI24REH (hybrid L24)^
^L1−1^	^F^	^6^	^c.595G>A^	^p.(Val199Met)^	^c.916G>A^	^p.(Ala306Thr)^	^4^	^2^	^Profound^	^Normal^	^−^	^+^	^Left^	^AB Clarion C−II, Hifocus−1^
^L1−2^		^7^					^5^		^Profound^			^+^	^Right^	^CI24RE (CA)^
^M1^	^F^	^47^	^c.208del^	^p.(his70fs)^	^c.1276G>A^	^p.(Ala426Thr)^	^41^	^6^	^Profound^	^Normal^	^−^	^+^	^Left^	^AB Clarion C−II, Hifocus−1^
^M2^	^M^	^47^	^c.208del^	^p.(his70fs)^	^c.1276G>A^	^p.(Ala426Thr)^	^35^	^12^	^Profound^	^Hyperreflexia^	^−^	^+^	^Left^	^AB Clarion C−II, Hifocus−1^
^M3^	^M^	^44^	^c.208del^	^p.(his70fs)^	^c.1276G>A^	^p.(Ala426Thr)^	^24^	^20^	^Profound^	^Normal^	^−^	^+^	^Left^	^AB HiRes 90 K Advantage, HiFocus Mid−Scala^
^M4^	^F^	^50^	^c.208del^	^p.(his70fs)^	^c.1276G>A^	^p.(Ala426Thr)^	^34^	^16^	^Profound^	^Hyperreflexia^	^−^	^−^	^Left^	^CI612^
^N1^	^M^	^51^	^c.413C>A^	^p.(Ala138Glu)^	^c.413C>A^	^p.(Ala138Glu)^	^41^	^10^	^Profound^	^Normal^	^−^	^+^	^Left^	^CI24M^
^O1^	^M^	^28^	^c.323−6G>A^	^p.(Val108fs)^	^c.413C>A^	^p.(Ala138Glu)^	^25^	^3^	^Profound^	^Hyperreflexia^	^−^	^+^	^Right^	^CI24RE (ST)^
^O2^	^M^	^30^	^c.323−6G>A^	^p.(Val108fs)^	^c.413C>A^	^p.(Ala138Glu)^	^26^	^4^	^Profound^	^Hyporeflexia^	^−^	^−^	^Left^	^CI512^
^P1^	^M^	^54^	^c.413C>A^	^p.(Ala138Glu)^	^c.413C>A^	^p.(Ala138Glu)^	^NA^	^NA^	^Profound^	^Normal^	^−^	^−^	^Right^	^CI24RE (CA)^
^Q1^	^M^	^10^	^c.46C>T^	^p.(Arg16*)^	^c.595G>A^	^p.(Val199Met)^	^2^	^8^	^Profound^	^NA^	^−^	^+^	^Right^	^Med−El concerto flex28^
^R1−1^	^M^	^7^	^c.916G>A^	^p.(Ala306Thr)^	^c.916G>A^	^p.(Ala306Thr)^	^2^	^6^	^Severe^	^NA^	^−^	^−^	^Right^	^CI532^
^R1−2^		^8^							^Severe^			^−^	^Left^	^CI632^
^S1^	^F^	^31^	^c.413C>A^	^p.(Ala138Glu)^	^c.916G>A^	^p.(Ala306Thr)^	^15^	^16^	^Profound^	^NA^	^−^	^+^	^Right^	^CI24REH (hybrid L24)^
^T1^	^M^	^62^	^c.413C>A^	^p.(Ala138Glu)^	^c.1276G>A^	^p.(Ala426Thr)^	^22^	^40^	^Profound^	^Normal^	^−^	^−^	^Left^	^AB HiRes 90 K Advantage, HiFocus Mid−Scala^
^U1^	^F^	^54^	^c.916G>A^	^p.(Ala306Thr)^	^c.316C>T^	^p.(Arg106Cys)^	^36^	^18^	^Profound^	^Normal^	^−^	^−^	^Left^	^AB HiRes 90 K Advantage, HiFocus Mid−Scala^
^V1^	^F^	^13^	^c.413C>A^	^p.(Ala138Glu)^	^c.208del^	^p.(his70fs)^	^1^	^12^	^Severe^	^NA^	^−^	^−^	^Left^	^CI632^
^W1^	^M^	^9^	^c.413C>A^	^p.(Ala138Glu)^	^c.916G>A^	^p.(Ala306Thr)^	^9^	^0^	^Severe^	^NA^	^−^	^−^	^Right^	^CI532^

*HL* hearing loss, *NA* not available, *AB* advanced bionics

^*^Patients C1, E1, H1, l1, M1-4, and O1-2 are previously described by Weegerink et al.

^**^cDNA and protein nomenclature is based on transcript NM_024022.4

^***^According to WHO’s grades of hearing impairment

^****^Tested with electronystagmography (ENG) which was performed with vestibular caloric, and rotary chair testing. Patient L1, and M1-M4 were only tested with the rotary chair test

**Table 3:** Pre-implantation pure tone average (PTA) and phoneme scores of the implanted ears in the TMPRSS3-group

	Pre-implantation PTA_0.5-4kHz_		Pre-implantation Phoneme score at 70 dB		CI-use post-implantation	
		PTA_0.5-4kHz_ (dB HL)^**^		Phoneme score at 70 dB (%)^**^	CI-user	Non-/limited CI-user
Hearing aid prior to implantation (N = 21, 64%)	Aided PTA (N = 21)	59 ± 16	Aided phoneme score (N = 14)	22 ± 27	20 (95%)	1 (5%)
No-hearing aid prior to implantation (N = 12, 36%)	Unaided PTA (N = 12)	82 ± 15	Unaided phoneme score (N = 19)	41 ± 26	9 (75%)	3 (25%)
Best-aided/unaided* (N = 33, 100%)	Best-aided/unaided* (N = 33)	67 ± 19	Best-aided/unaided* (N = 33)	33 ± 28	29 (88%)	4 (12%)

PTA indicates pure tone average; CI, cochlear implant

^*^The best-aided/unaided scores were calculated from aided scores from patients using hearing aids prior to implantation in combination with the unaided scores from patients not using hearing aids prior to implantation. When aided scores from patients using hearing aids were not available, unaided scores were also used

^**^PTA_0.5-4kHz_ and phoneme scores are displayed as mean ± standard deviation

### Audiological tests

**Fig. 1:**
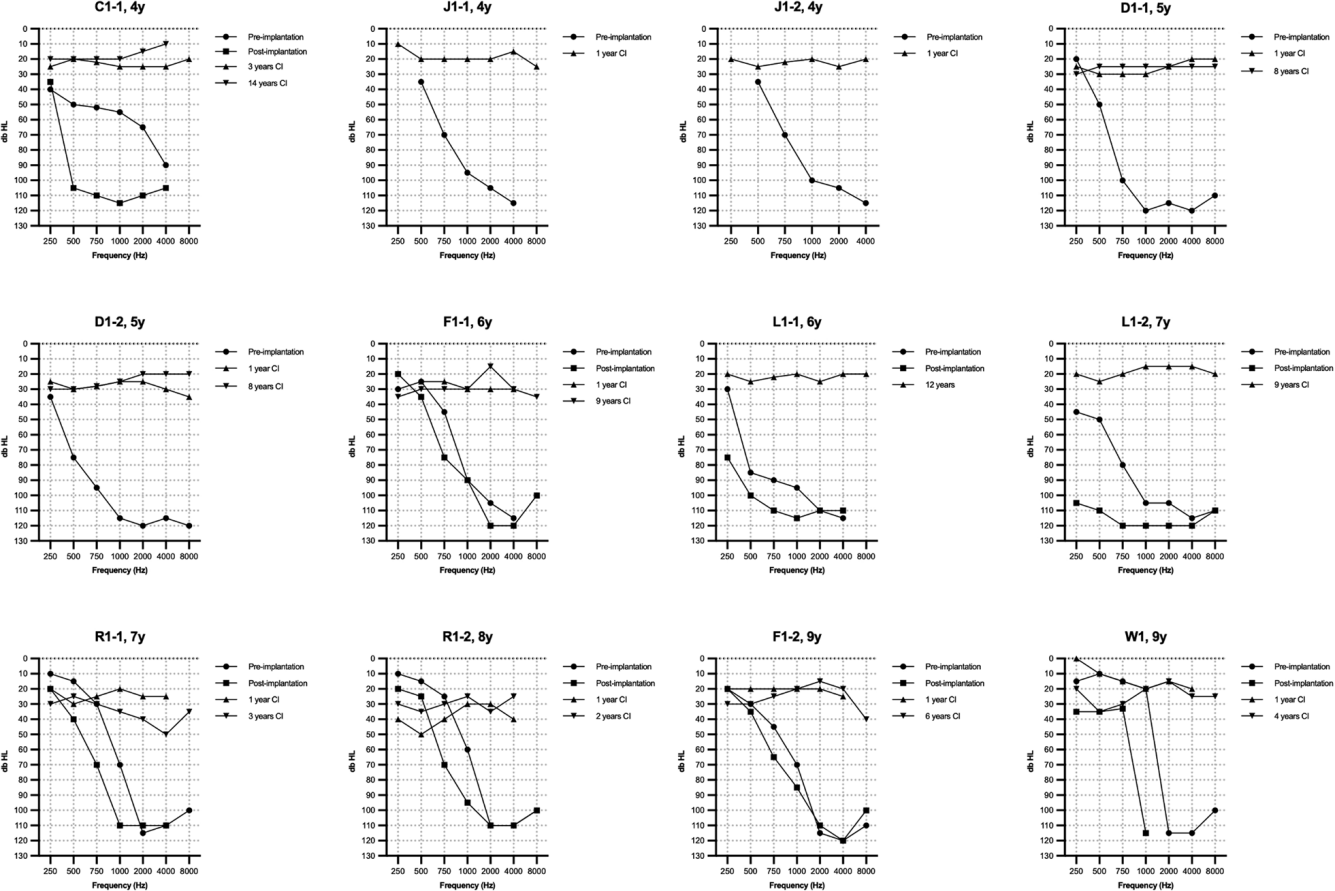

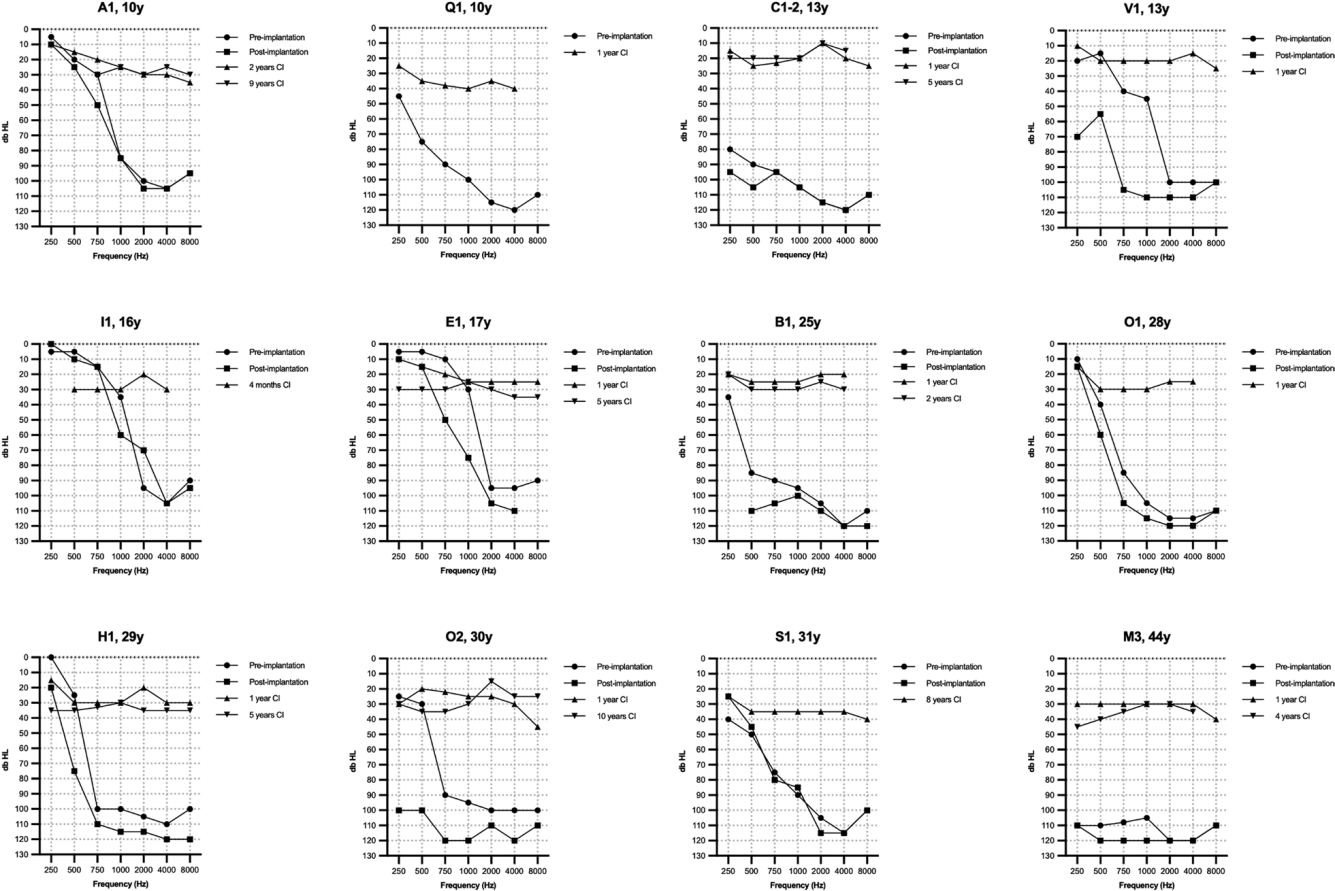

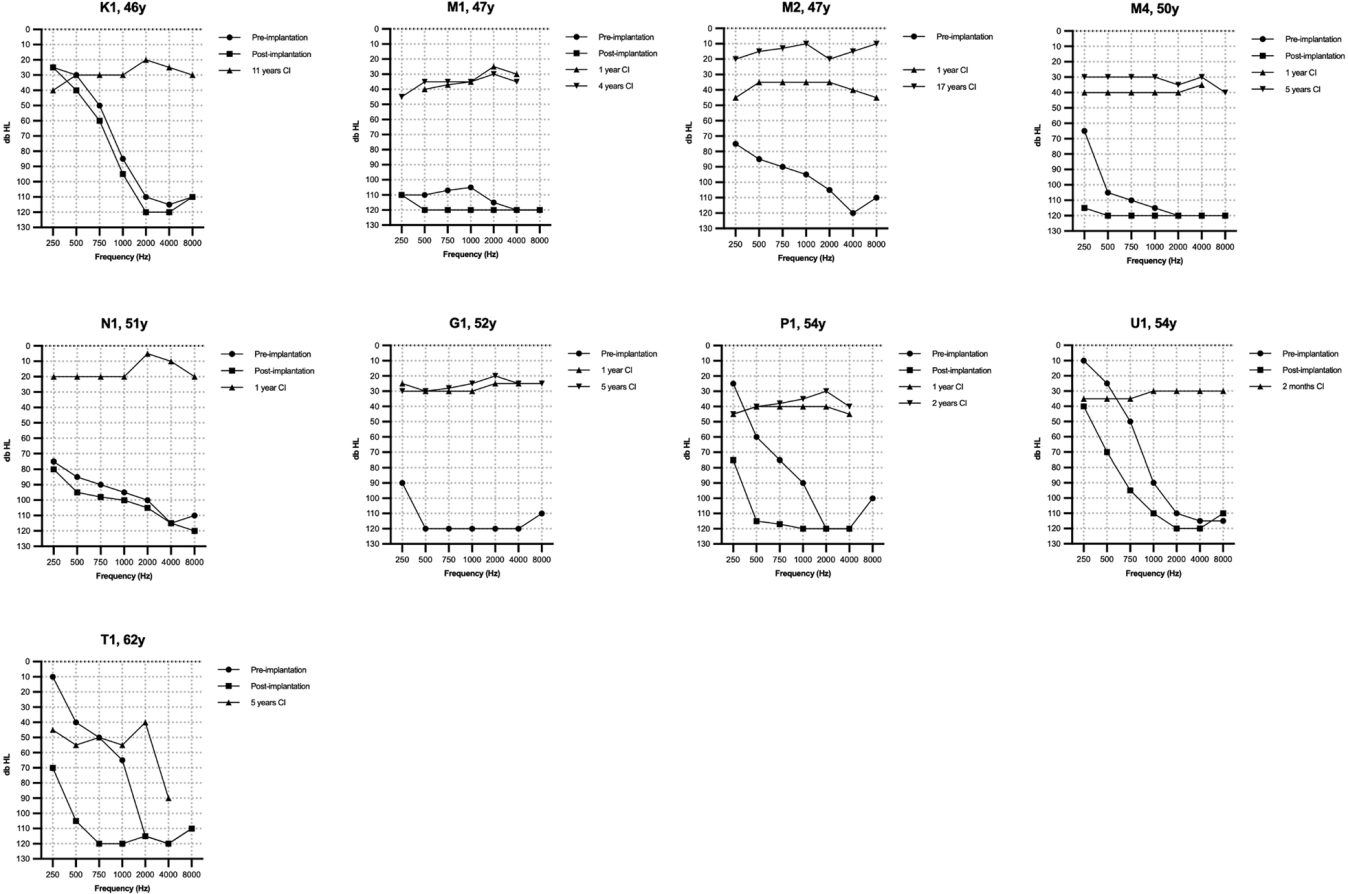
Audiograms in the* TMPRSS3*-group. Audiograms are ranged from lowest to highest age during implantation. Pre-implantation audiograms indicate unaided audiograms. Post-implantation audiograms were measured at 6 ± 5 months post implantation

**Fig. 2:**
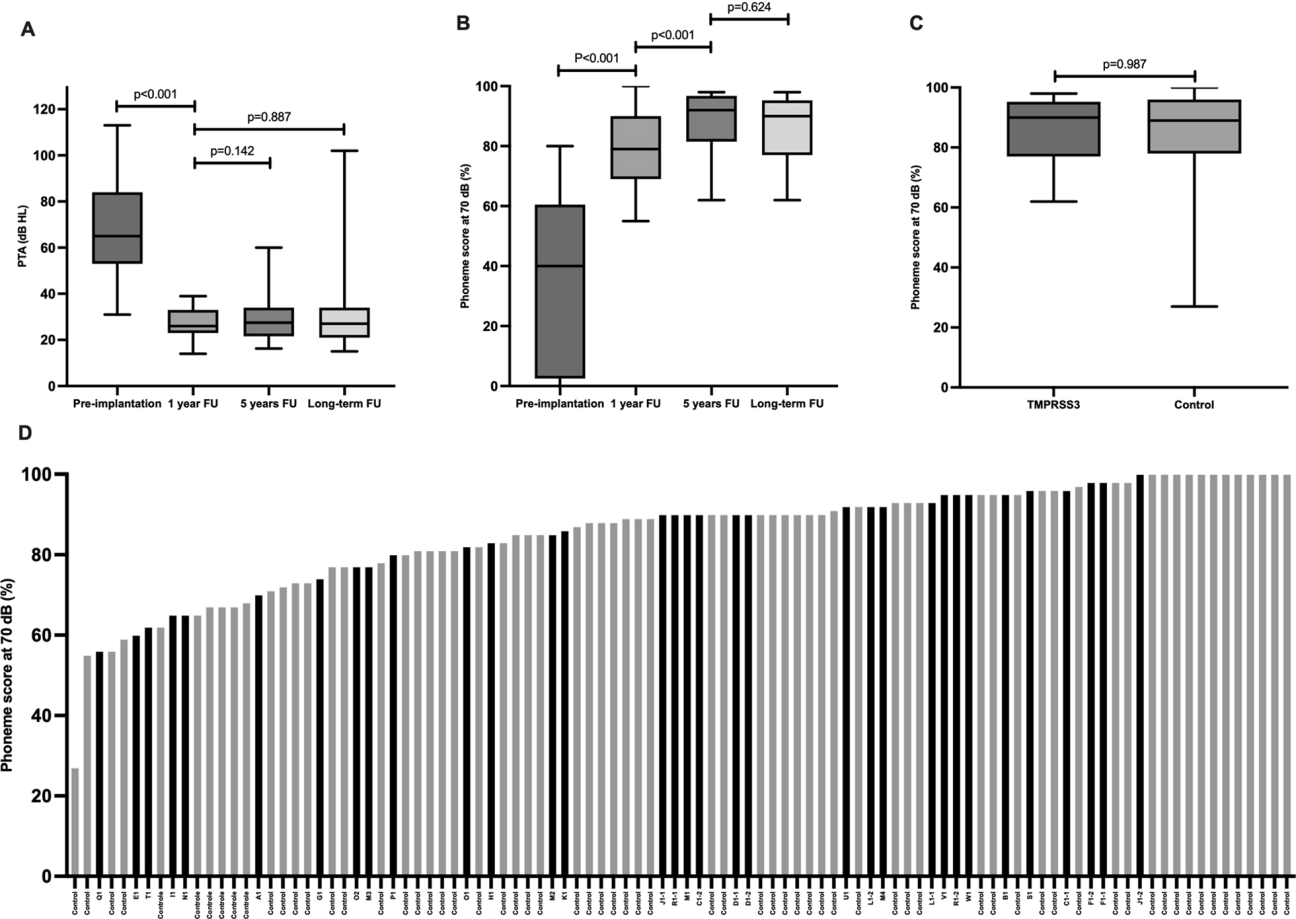
*Cochlear implant performance in TMPRSS3*- and control-group.** A** Boxplot of pure tone average (PTA) scores in the TMPRSS3-group. The pre-implantation PTA indicates the best-aided/unaided PTA measured with inserts/headphone. The follow-up PTA are free field measurements. The long-term follow up was 7.2 ± 3.7 years. **B** Boxplot of phoneme scores at 70 dB in the *TMPRSS3*-group. The pre-implantation phoneme-score indicates the best-aided/unaided phoneme score measured with inserts/headphone. The follow-up phoneme scores are free field measurements. The long-term follow up was 9.8 ± 3.7 years. **C** Boxplot of the long-term phoneme scores at 70 dB in the *TMPRSS3*-group and control-group, with a follow up time of respectively 9.8 ± 3.7 and 8.7 ± 3.2 years. **D** Phoneme score at 70 dB of the total study population (*TMPRSS3*- and control group) ranged from lowest to highest with a mean phoneme score of 85 ± 14% at a mean follow up time of 7.8 years. Black bars indicate the *TMPRSS3*-patients

Figure 1 shows the unaided pure tone audiogram of all implanted ears prior to cochlear implantation; in most subjects, a characteristic ski-slope configuration can be seen where thresholds are relatively preserved at low frequencies and severely affected at the higher frequencies. The preoperative unaided PTA_0.5-4kHz_ was 90 ± 17 dB HL (N = 33 ears), and this increased to 99 ± 16 dB HL (N = 26 ears) at 6 ± 5 months postoperatively (*p* < 0.001). There was no significant difference in HP% between the different surgical approaches (*p* = 0.273). We compared two TMPRSS3 groups of subjects who underwent cochlear implantation; one group had previously used a hearing aid, and the other group had not used a hearing aid as their residual hearing was sufficient. (Table 3). To enable a single pre- and postoperative comparison in terms of threshold and speech perception, we combined the best-aided and unaided results and compared them to the postoperative results. Table 3 also highlights the groups separately. The best-aided/unaided preoperative PTA_0.5–4kHz_ was 67 ± 19 dB HL (N = 33 ears, Table 3). One year after implantation, the postoperative PTA_0.5-4kHz_ significantly improved to 27 ± 7 dB HL (*p* < 0.001; N = 27) and remained stable over time (Fig. 2A).

**Fig. 3:**
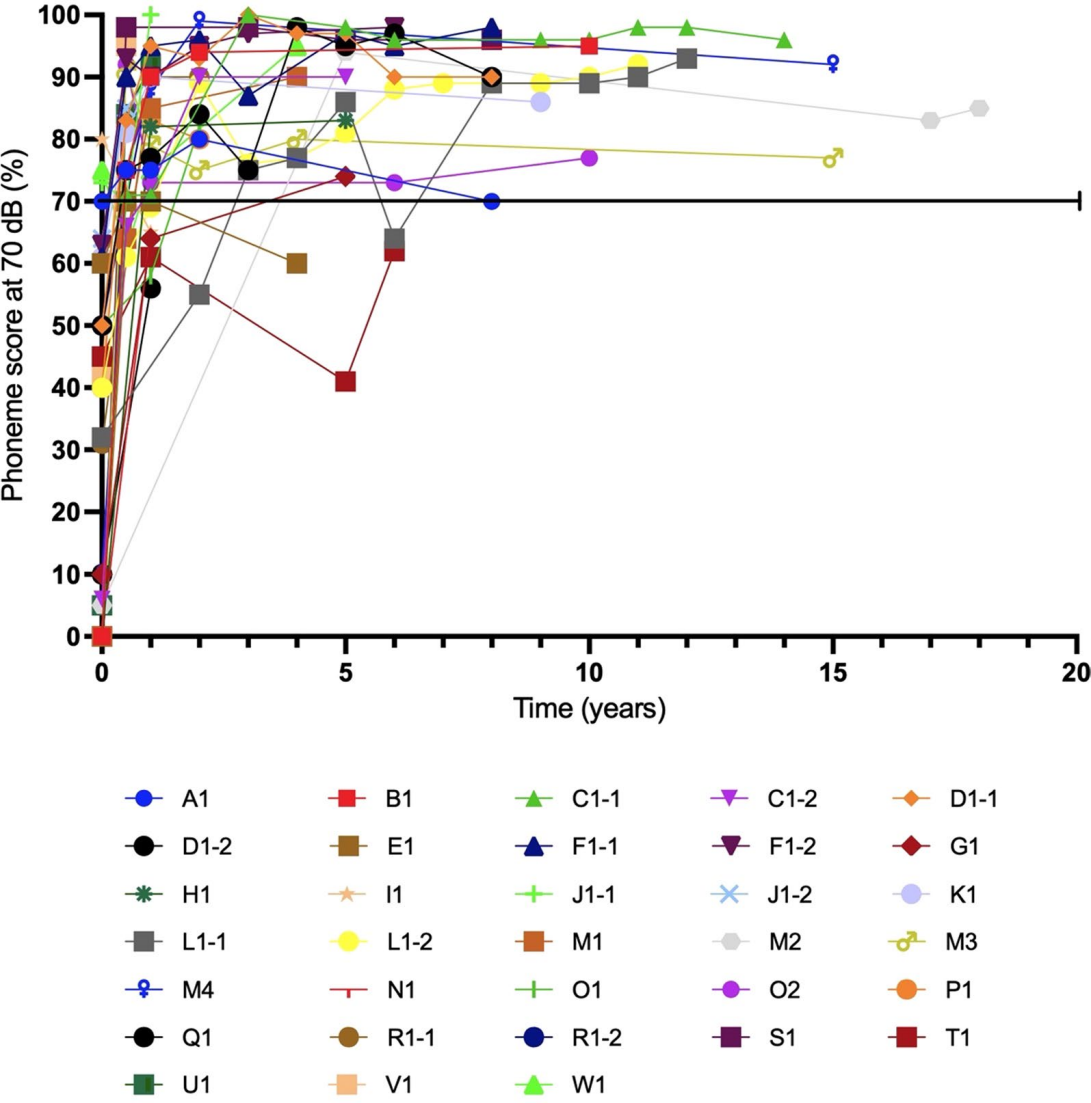
Postimplantation phoneme scores at 70 dB of each ear in the *TMPRSS3*-patients over the years

The average best-aided/unaided preoperative phoneme score at 70 dB was 33 ± 28% (Table 3). After a mean follow-up of 13 ± 3 months post-implantation, the average phoneme scores significantly increased to 79 ± 13% (*p* < 0.001; N = 31 ears), and further improved to 89 ± 10% at 4.9 ± 0.8 years post-implantation (*p* < 0.001, N = 16 ears, comparison 13 months vs 4.9 years), which remained stable after a mean follow-up of 9.8 ± 3.7 years with 86 ± 10% (*p* = 0.624, N = 18 ears) (Figs. 2B, 3). There was no significant difference in phoneme scores between the different surgical approaches (*p* = 0.401).

In the control group, the average phoneme score at 70 dB was 81 ± 21% (N = 49) 5 years after implantation, which remained stable at 85 ± 14% (N = 67) after a long-term follow-up of 8.7 ± 3.2 years. No significant differences were found between the control and the *TMPRSS3* group, both at 2 and 9 years after implantation, *p* = 0.830 and *p* = 0.987, respectively (Fig. 2C, D).

### Poor performers

As shown in Figs. 2D and 3, six subjects had a phoneme score of ≤ 70% and were evaluated as poor performers in more detail. Four of them (A1, E1, I1, and Q1) were implanted during childhood, but became limited or non-users of the CI post-implantation as they perceived no benefit. Three of these subjects (A1, E1, and I1) had high functional low frequency residual hearing pre-implantation (Fig. 1) and did not use hearing aids pre-implantation due to the absence of subjective benefit (Table 3). Two of these three subject preserved their residual hearing post-implantation (A1 and I1 with an HP% of respectively 95% and 98%, respectively) while the third had partial preservation (E1 with a HP% of 69%). Over time, the low frequency residual hearing of two subjects (A1 and E1) deteriorated, resulting in reusing their CI. Unfortunately, no phoneme scores after these re-starts are available.

The fourth limited-user (Q1) had limited residual hearing pre-implantation in the low frequencies. Unfortunately, the unaided audiogram post-implantation was unavailable. This subject also faced additional personal challenges and experienced learning difficulties that negatively influenced the performance of the CI.

The other two poor performers (N1 and T1) were implanted later in life, at 51 and 62 years, respectively. N1 had a phoneme score of 65% at 70 dB twelve months after implantation. Nevertheless, the subject reported a significant improvement in speech recognition and can converse on the telephone and in online meetings.

Subject T1 struggled to get used to high-pitched sounds because of his long-lasting high-frequency SNHL. Over the years, different processor settings were tried, including switching off the basal electrodes with or without less amplifying power of the other electrodes. Despite the phoneme score of 62% six years after implantation, subject T1 reported being satisfied with the current speech recognition.

### Age at implantation

Univariate regression analyses were performed to test whether the age at implantation and residual hearing post-implantation factors correlated with CI performance in the *TMPRSS3*-group. The univariate regression analysis, shown in Additional file 2: Fig. S1, shows that non-/limited CI-use was significantly correlated with more residual hearing post-implantation (R2 = 0.400, F = 16.03, *p* < 0.001). Also, older age at implantation was significantly associated with a lower postoperative phoneme score (i.e., the last-available score; R2 = 0.470, F = 23.9, *p* < 0.001).

A multiple regression analysis was performed to further study this second correlation while correcting for confounders including degree of hearing loss pre-implantation (i.e., unaided PTA_0.5-4kHz_), residual hearing, gender, and the use of hearing aids prior to implantation. The self-reported duration of SNHL was excluded from this analysis due to collinearity with the age at implantation (*r*(25) = 0.897, *p* < 0.001). After correcting for these confounders, older age at implantation was still significantly associated with a lower postoperative phoneme score (R2 = 0.893, F = 11.9, *p* < 0.001).

### Choice of electrode array

A total of 13 different electrode types were implanted in the *TMPRSS3-*group. Two subjects (S1 and K1) received a hybrid-L electrode array (Cochlear CI23REH). Both subjects had a mean phoneme score of 91 ± 7% at a mean follow-up time of 8.5 years after implantation. This was not significantly higher than 16 subjects with a non-hybrid implant showing a mean phoneme score of 86 ± 11% (*p* = 0.549). Both subjects lost residual hearing in the lower frequencies over the years, while their aided PTA and phoneme score at 70 dB remained stable, with an unknown contribution from the acoustic component (Fig. 3).

All implanted electrode arrays in the *TMPRSS3*-group were classified and grouped as either a lateral wall electrode (LWE; N = 15, 45%) or a peri-modiolar electrode (PME; N = 18, 55%), see, e.g., Adiitional file 1: Table 1. At 1-year post-implantation, no difference (*p* = 0.594) was found between the groups, with an average phoneme score at 70 dB of 77 ± 15% and 80 ± 12% for the LWE and PME groups. Also, at the longest follow-up measurement of 10 years post-implantation, no significant difference was found between the groups (i.e., a phoneme score of 89 ± 9% and 83 ± 12% for LWE and PME, respectively; *p* = 0.360).

### Genotype–phenotype correlation

Six missense and five truncating variants in *TMPRSS3* were identified in the study population, as shown in Table 4. The truncating variant c.936del (p.Pro313fs) was not previously described in literature. This variant was classified as likely pathogenic because it is a truncating variant not detected in control populations (GnomAD v2.1.1).

**Table 4:** *TMPRSS3* variants in study population

	Transcript	cDNA	Protein	domain	Variant type	Classification	References
M1	NM_024022.4	c.46C>T	p.(Arg16*)	*	Truncating	Pathogenic	[37]
M2	NM_024022.4	c.208del	p.(his70fs)	*	Truncating	Pathogenic	[38]
M3	NM_024022.4	c.280G>T	p.(Gly94*)	LDLRA	Truncating	pathogenic	[39]
M4	NM_024022.4	c. 316C>T	p.(Arg106Cys)	Serine protease	Missense	(Likely) pathogenic	[39]
M5	NM_024022.4	c.323-6G>A	p.(Val108fs)	LDLRA	Truncating	(Likely) pathogenic	[4]
M6	NM_024022.4	c.325C>T	p.(Arg109Trp)	LDLRA	Missense	Pathogenic	[40]
M7	NM_024022.4	c.413C>A	p.(Ala138Glu)	SRCR	Missense	(Likely) pathogenic	[41]
M8	NM_024022.4	c.595G>A	p.(Val199Met)	SRCR	Missense	(Likely) pathogenic	[9]
M9	NM_024022.4	c.916G>A	p.(Ala306Thr)	Serine protease	Missense	(Likely) pathogenic	[42]
M10	NM_024022.4	c.936del	p.(Pro313fs)	Serine protease	Truncating	Likely pathogenic	**
M11	NM_024022.4	c.1276G>A	p.(Ala426Thr)	Serine protease	Missense	(Likely) pathogenic	[43]

^*^Variant is not located in a domain

^**^Variant is not previously described in literature

Four different truncating variants were found in the study population, but no subjects with biallelic truncating variants could be identified. No correlation was found between the phenotype (self-reported age of onset and degree of hearing loss) and the variant type (results not shown). The found variants in *TMPRSS3* affected three different domains, including LDLRA, SRCR, and Serine protease (see Table 4). There was also no correlation between the affected domains and the corresponding phenotype (results not shown).

## Discussion

This study showed that CI recipients with *TMPRSS3-*associated SNHL showed favourable and consistent outcomes in both short- and long-term follow-up evaluations. These results were comparable to those obtained in a control group with genetic postlingual SNHL. These findings are in line with three recent literature reviews, which evaluated CI performance in this population with shorter follow-up times and more heterogeneous outcome measures [3, 13, 14]. Our study, therefore, provides further evidence to support the strong recommendation of CI for hearing rehabilitation in subjects with *TMPRSS3-*associated SNHL. Despite beneficial outcomes, there were six subjects with less beneficial outcomes. This included some children in puberty with sufficient residual hearing post implantation, which complicated rehabilitation. In addition, implantation in two patients at an older age, and therefore a longer duration of hearing loss, negatively influenced CI outcomes as well.

In addition, we found that a relatively high proportion of subjects (36%) did not use hearing aids prior to implantation, mainly due to absence of subjective benefit. This lack of usage may be attributed to the typical ski-slope high-frequency hearing loss associated with *TMPRSS3-*related SNHL. Existing hearing aids may not provide sufficient amplification of the mid-to-high frequencies required for speech perception, leading to poor outcomes [24]. Additionally, previous research has suggested that high-frequency amplification may not sufficiently improve speech perception due to the suprathreshold issues caused by cochlear hearing loss [25].

### TMPRSS3 and SGN involvement

The second aim of this study was to evaluate the spiral ganglion hypothesis proposed by Eppsteiner et al. using the *TMPRSS3-*group. This hypothesis suggests that the spiral ganglion cells play a significant role in auditory processing of individuals with *TMPRSS3* variants who received a cochlear implant. According to this hypothesis, pathogenic variants in genes preferentially expressed in the SGN, such as *TMPRSS3*, may lead to poor CI performance [8].

In the study by Shearer et al., *TMPRSS3-*associated hearing loss led to poor CI performance in subjects with poor auditory nerve neurophonics (ANN), but intact cochlear microphonics (CMs), indicating SGN loss [7]. However, our study showed that subjects with *TMPRSS3*-associated SNHL who received cochlear implants achieve good long-term performance, equally to the control-group. This suggests that either TMPRSS3’s involvement in SGN may not be as significant as previously thought, or that the spiral ganglion hypothesis is incorrect. These results are consistent with studies demonstrating limited Tmprss3-expression in SGNs in mice [14, 26]. In human inner ear organoids, TMPRSS3 expression is mostly limited to HCs [13], which confirms limited SGN involvement in *TMPRSS3-*associated SNHL and supports the good long-term performance observed in our study. While these results do not entirely rule out the possibility of a general SGN hypothesis, evidence from mouse models and expression patterns in human inner ear organoids suggests that SGN involvement in *TMPRSS3* is unlikely. Additional studies are needed in genotyped CI recipients with affected genes that are expressed in the SGN to confirm or refute this hypothesis.

### Poor performers

Despite the overall good CI outcomes in subjects with *TMPRSS3*-related SNHL, in six CI recipients, CI performance remained behind. Poor performance was observed in subjects with high levels of residual hearing in the lower frequencies. These subjects had difficulty adapting to the sound of their CI, resulting in limited or non-use of the CI. Three of these subjects did not use hearing aids prior to implantation. In two subjects SNHL increased over time which ultimately led them to becoming CI users. The same is expected to apply to the other two in due time. These findings indicate that CI might be too early in children with high functional residual hearing in the lower frequencies without the subjective benefit of hearing aids prior to implantation.

Additionally, poor performance was significantly correlated with an older age at the time of implantation. This is likely because older age at implantation is often associated with a more extended period of lack of auditory stimulation, especially in the high frequencies of subjects with *TMPRSS3*. This was also likely the case in the two poor performers in the study of Eppsteiner et al. [8], and Shearer et al. [7]. Both factors, older age at implantation, and longer duration of SNHL, have previously been negatively correlated to poor outcomes in post-lingually adult CI-recipients [27].

### Choice of electrode array

The Hybrid-L electrode was developed as a shorter straight electrode to facilitate electrical and acoustic stimulation by preserving low-frequency hearing. Recipients with these electrodes had increased speech recognition compared to electric stimulation only [28]. Although most subjects in the present study had preserved low-frequency hearing thresholds, only two received a CI with a Hybrid-L electrode. This is likely related to the general progressive nature of *TMPRSS3*-associated hearing loss, leading to a choice for a longer electrode to stimulate low-frequencies. Both subjects had good CI performance, with an unknown contribution from the acoustic component, but were not significantly better than the other CI recipients. In the study by Shearer et al., all three *TMPRSS3* subjects were implanted with a hybrid electrode. Two of them had poor outcomes, of whom one did not use the acoustic component due to no measurable residual hearing at 500 Hz [7].

We found no significant difference in speech recognition or HP between LWE and PME electrodes. This is in line with previous inconclusive or contradictory studies regarding the position of the CI electrode close to the modiolus (PME) or following the lateral wall (LWE), and its effect on CI performance [29–31]. Additionally, the surgical approach had no significant impact on CI performance nor on HP as was previously found [32, 33]. The findings in this study, although based on a small number of subjects, suggest that neither the type of electrode nor the surgical approach seems to influence CI performance in subjects with *TMPRSS3-*associated SNHL.

### Genotype–phenotype correlation

Locus DFNB8 was identified as a disease locus for hearing loss in a family with post-lingual progressive SNHL in 1996 [34]. In the same year, another research group independently identified locus DFNB10 in a family with profound SNHL, including one-week-old twin girls [35]. Later, Scott et al. found that both loci were located on the same gene (*TMPRSS3).* Additionally, they concluded the mutation in the DFNB8 family allowed some regular protein expression in contrast to the mutation in the DFNB10-family, accounting for the phenotypic difference between the two families [4]. Ever since, *TMPRSS3-*associated SNHL has been presumed to present with either profound prelingual SNHL (DFNB10) or postlingual, progressive SNHL (DFNB8) [9].

In 2021, Moon et al. proposed that the combination of a missense variant and a truncating variant resulted in DFNB8, whereas two truncating (or loss-of-function) pathogenic variants led to DFNB10 [3]. The present study provides no evidence for specific truncating or non-truncating variant combinations that lead to a particular (more or less severe) phenotype. Also, a correlation between the affected domains and the phenotype could not be found. Multi-centre studies on larger numbers of subjects are needed to elucidate this correlation further.

### Strengths and limitations

This is the first study evaluating CI performance in subjects with *TMPRSS3*-associated SNHL at short- and long-term follow-up. Furthermore, to our knowledge, this is the largest study population in which CI performance is evaluated in patients with *TMPRSS3*-associated SNHL.

The main limitation of this study is the retrospective design, which inevitably leads to missing data. Furthermore, the control group differed significantly from the *TMPRSS3* group on two factors. Firstly, the control group included subjects with EVAs. Since these subjects have progression of SNHL in the same age category as the *TMPRSS3-*group, we did not want to exclude these subjects from the control group. We do not think the EVAs in the control group influenced the CI performance because previous studies showed that the outcomes in pediatric CI recipients with EVA are (broadly) comparable to results in pediatric CI recipients without inner ear malformations [19, 20]. Also, the surgical success and major complication rates in subjects with EVA are similar to studies in the general CI population [21].

Secondly, a significant difference was found in the surgical approach between the *TMPRSS3*- and the control group. Since subjects with *TMPRSS3-*associated hearing loss have, in general, sufficient residual hearing in the lower frequencies, the round window approach was more frequently used since this technique is supposed to lead to better HP. However, a systematic review comparing the cochleostomy with the round window approach showed no benefit of one surgical procedure over the other regarding HP [36]. Moreover, the present study found no significant difference in the phoneme scores or HP in the different surgical approaches. Therefore, we believe the surgical approach did not influence the CI performance.

## Conclusion

In summary, CI-recipients with *TMPRSS3*-associated SNHL have an adequate outcome at both short- and long-term follow-up. Some subjects with residual hearing post-implantation or older age at implantation exhibited less favourable outcomes. Therefore, we would recommend not to wait too long with CI in adults. For children with poor low frequency thresholds pre-implantation, we recommend early implantation. However, in children with near-normal low frequency thresholds pre-implantation, specific preoperative counseling on potential difficulties during rehabilitation is required when residual hearing persists, especially in children who are in puberty. The type of electrode or surgical approach does not influence CI performance in subjects with *TMPRSS3-*associated SNHL. Furthermore, we identified a new likely pathogenic variant in *TMPRSS3*: c.936del (p.Pro33fs). Finally, since TMPRSS3 is mainly expressed in the HCs, we could not confirm nor refute the spiral ganglion hypothesis.

### Supplementary Information

**Additional file 1: Table S1**. Classification of the implanted electrode types in the TMPRSS3-groep.

**Additional file 2: Fig. S1**. Univariate logistic regressions in TMPRSS3-Patients

## Data Availability

The datasets used and analyzed during the current study are available from the corresponding author on reasonable request.

## Abbreviations

ANN: Auditory nerve neurophonics
CI: Cochlear implant
CM: Cochlear microphonics
CMV: Cytomegalovirus
dB: Decibel
ENG: Electronystagmography
EVA: Enlarged vestibular aqueduct
HC: Hair cell
HP: Hearing preservation
HP%: Percentage of residual hearing preservation
HSV: Herpes simplex virus
LWE: Lateral wall electrode
PME: Peri-modiolar electrode
PTA: Pure tone average
SGN: Spiral ganglion neuron
SNHL: Sensorineural hearing loss
vHIT: Video head impulse test
