# Supplementary material for: Stable long-term outcomes after cochlear implantation in subjects with TMPRSS3 associated hearing loss: a retrospective multicentre study
Source: J Otolaryngol Head Neck Surg. 2023 Dec 15;52:82. doi: 10.1186/s40463-023-00680-3 (PMC10724910; doi:10.1186/s40463-023-00680-3)
Supplement: Supplementary file 1 — Additional file 1: Table S1. Classification of the implanted electrode types in the TMPRSS3-groep. [file 40463_2023_680_MOESM1_ESM.docx]

**Supplementary table 1.** Classification of the implanted electrode types in the *TMPRSS3*-groep

|  | *Type of electrode* | Implanted (N) |
| --- | --- | --- |
| AB Clarion CII, HiFocus-1 | LWE | 3 |
| AB HiRes 90K Advantage, HiFocus Mid-Scala | PME | 3 |
| Cochlear CI24M | LWE | 1 |
| Cochlear CI24RE (CA) | PME | 5 |
| Cochlear CI24RE (ST) | LWE | 5 |
| Cochlear CI24REH (hybrid L24) | LWE | 2 |
| Cochlear CI422 | LWE | 1 |
| Cochlear CI512 | PME | 2 |
| Cochlear CI522 | LWE | 2 |
| Cochlear CI532 | PME | 2 |
| Cochlear CI612 | PME | 1 |
| Cochlear CI632 | PME | 5 |
| Med-El concerto Pin flex28 23.8 | LWE | 1 |

PME indicates peri-modiolar electrode array; LWE, Lateral wall electrode array.
