# Supplementary material for: Stable long-term outcomes after cochlear implantation in subjects with TMPRSS3 associated hearing loss: a retrospective multicentre study
Source: J Otolaryngol Head Neck Surg. 2023 Dec 15;52:82. doi: 10.1186/s40463-023-00680-3 (PMC10724910; doi:10.1186/s40463-023-00680-3)
Supplement: Supplementary file 2 — Additional file 2: Fig. S1. Univariate logistic regressions in TMPRSS3-Patients [file 40463_2023_680_MOESM2_ESM.docx]

**Supplementary Figure 1. Univariate logistic regressions in TMPRSS3-Patients**

| ***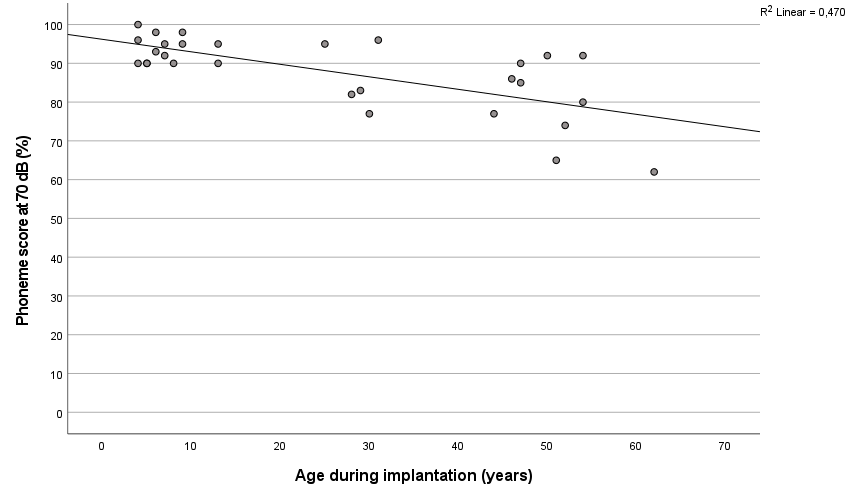*A** | **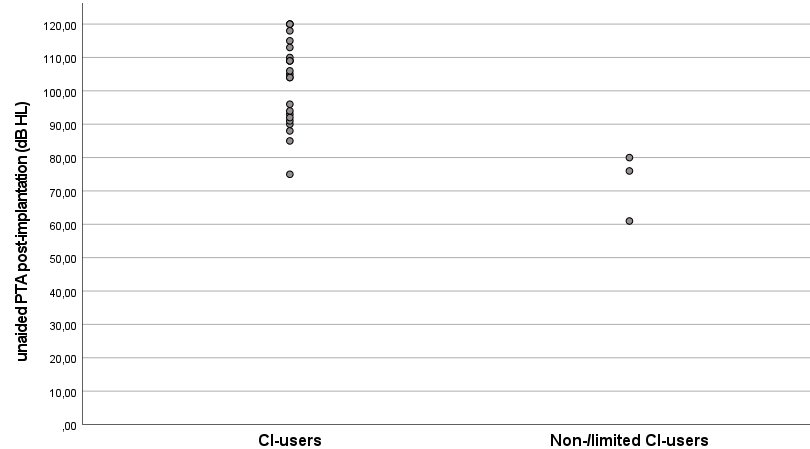B** |
| --- | --- |

**A.** Univariate regressions with the last available phoneme score at 70 dB as dependent variable with a mean follow-up time of 6.7 years, and the age during implantation as independent variable with a mean of 26 years in 29 ears; R^2^= 0.470, F=23.9, P<0.001. **B.** Univariate regression with the unaided PTA_0.5-4kHz_ post-implantation measured within <1 year post implantation as dependent variable with a mean follow-up time of 6±5 months, and CI-use as independent variable; R^2^=0.400, F=16.03, p<0.001.
The four limited-/non-users (A1, E1, I1, Q1) were excluded from the first analysis (A), since their data are not representative, because previous research showed that daily CI processor utilization of at least 12 hours/day is needed to achieve optimal speech recognition performance for most subjects ^1^.

^1^ Lindquist NR, Dietrich MS, Patro A, Henry MR, DeFreese AJ, Freeman MH, Perkins EL, Gifford RH, Haynes DS, Holder JT. Early Datalogging Predicts Cochlear Implant Performance: Building a Recommendation for Daily Device Usage. Otol Neurotol. 2023 Aug 1;44(7):e479-e485.
